# Exosomes derived from human placental mesenchymal stem cells ameliorate myocardial infarction via anti-inflammation and restoring gut dysbiosis

**DOI:** 10.1186/s12872-022-02508-w

**Published:** 2022-02-17

**Authors:** Libo Yang, Ting Wang, Xiaoxia Zhang, Hua Zhang, Ning Yan, Guoshan Zhang, Ru Yan, Yiwei Li, Jingjing Yu, Jun He, Shaobin Jia, Hao Wang

**Affiliations:** 1 Clinical Medical College, Ningxia Medical University, Yinchuan, 750004 Ningxia China; 2 Heart Centre and Department of Cardiovascular Diseases, General Hospital of Ningxia Medical University, Yinchuan, 750004 Ningxia China; 3 Ningxia Key Laboratory of Vascular Injury and Repair Research, Ningxia Medical University, Yinchuan, 750004 China; 4 Department of Pathogenic Biology and Medical Immunology, School of Basic Medical Sciences, Ningxia Medical University, Yinchuan, 750004 Ningxia China; 5 College of Traditional Chinese Medicine, Ningxia Medical University, Yinchuan, 750004 Ningxia China; 6 Department of Beijing National Biochip Research Center Sub-Center in Ningxia, General Hospital of Ningxia Medical University, Yinchuan, China

**Keywords:** MI, PMSC-Exos, Anti-inflammation, Gut microbiota, SCFAs, LPS

## Abstract

**Background:**

Myocardial infarction (MI) represents a severe cardiovascular disease with limited therapeutic agents. This study was aimed to elucidate the role of the exosomes derived from human placental mesenchymal stem cells (PMSCs-Exos) in MI.

**Methods:**

PMSCs were isolated and cultured in vitro, with identification by both transmission electron microscopy (TEM) and nanoparticle tracking analysis (NTA). To further investigate the effects of PMSC-Exos on MI, C57BL/6 mice were randomly divided into Sham group, MI group, and PMSC-Exos group. After 4 weeks of the intervention, cardiac function was assessed by cardiac echocardiography, electrocardiogram and masson trichrome staining; lipid indicators were determined by automatic biochemical instrument; inflammatory cytokines were measured by cytometric bead array (CBA); gut microbiota, microbial metabolites short chain fatty acids (SCFAs) as well as lipopolysaccharide (LPS) were separately investigated by 16S rRNA high throughput sequencing, gas chromatography mass spectrometry (GC–MS) and tachypleus amebocyte lysate kit; transcriptome analysis was used to test the transcriptional components (mRNA\miRNA\cirRNA\lncRNA) of PMSC-Exos.

**Results:**

We found that human PMSC-Exos were obtained and identified with high purity and uniformity. MI model was successfully established. Compared to MI group, PMSC-Exos treatment ameliorated myocardial fibrosis and left ventricular (LV) remodeling (*P* < 0.05). Moreover, PMSC-Exos treatment obviously decreased MI molecular markers (AST/BNP/MYO/Tn-I/TC), pro-inflammatory indicators (IL-1β, IL-6, TNF-α, MCP-1), as well as increased HDL in comparison with MI group (all *P* < 0.05). Intriguingly, PMSC-Exos intervention notably modulated gut microbial community via increasing the relative abundances of *Bacteroidetes*, *Proteobacteria*, *Verrucomicrobia*, *Actinobacteria*, *Akkermansia*, *Bacteroides*, *Bifidobacterium*, *Thauera and Ruminiclostridium*, as well as decreasing *Firmicutes* (all *P* < 0.05), compared with MI group. Furthermore, PMSC-Exos supplementation increased gut microbiota metabolites SCFAs (butyric acid, isobutyric acid and valeric acid) and decreased LPS in comparison with MI group (all *P* < 0.05). Correlation analysis indicated close correlations among gut microbiota, microbial SCFAs and inflammation in MI.

**Conclusions:**

Our study highlighted that PMSC-Exos intervention alleviated MI via modulating gut microbiota and suppressing inflammation.

## Background

Myocardial infarction (MI) represents a severe cardiovascular disease, characterized by coronary ischemia and associated myocardial necrosis [1], contributing to the pathological ventricular remodeling and ventricular dysfunction and heart failure [2]. Although the effective revascularization can attenuate MI, more than 7% mortality and 22% morbidity still occur in a year [3, 4]. Thus, there remains a clear and unmet need for the identification of novel therapeutic strategies for MI.

Upon MI, massive death of cardiomyocytes triggers a strong inflammatory response with the main manifestation of higher concentrations of interleukin (IL)-6, IL-1β, monocyte chemoattractant protein-1 (MCP-1) and tumor necrosis factor α (TNF-α) [5, 6]. Anti-TNF-α therapy dramatically decreased inflammatory cytokine levels, inflammatory cell infiltration in and around the infarct area [5]. A survey has suggested that fibroblasts exert pro-inflammatory and matrix-degrading effects in the early inflammation phase of MI [7]. Thus, anti-inflammation is a critical approach in improving cardiac injury and left ventricular (LV) remodeling.

Accumulating studies have shown that by injuring the integrity and permeability of gut barrier, the gut dysbiosis aggravates the translocation of lipopolysaccharide (LPS) into circulation to trigger inflammation cascade through the LPS-TLR4 pathway, indicating that gut microbiota is inextricably linked to the occurrence of inflammation [8–10]. A clinical study demonstrated that atherosclerotic cardiovascular disease individuals possessed high quantities of *Enterobacteriaceae*, *Streptococcus* and *Eggerthella lenta* in comparison with healthy people [11]. Consistently, animal experiments revealed that the abundances of *Synergistetes*, *Spirochaetes*, *Lachnospiraceae*, *Syntrophomonadaceae* and *Tissierella* in MI model group were higher than sham group [12]. Moreover, probiotic *Lactobacillus rhamnosus* GR-1 administration attenuated LV hypertrophy and heart failure after MI in rats [13]. In addition to LPS, other intestinal microbiota derived metabolites such as short chain fatty acids (SCFAs) have been demonstrated to exhibit an anti-inflammation role by inhibiting macrophage activation via binding to G protein-coupled receptors (GPRs) or histone deacetylase (HDAC) inhibition [14, 15]. Additionally, SCFAs have been shown to lower risk factors of cardiovascular disease (CVD), including the reduction of blood pressure and the regulation of glucose/lipid homeostasis [16]. Taken together, the understanding of complicated impacts of gut microbiota and their metabolites on the MI may contribute to a novel therapeutic approach [15].

Exosomes, vesicular bodies secreted by cells to the extracellular space, as an important carrier of biological information for facilitating intercellular communication, have been demonstrated to participate in the pathophysiological process of CVD [1, 17]. Recently, the effects of exosomes in the inflammatory reaction and immune regulation in MI have been gradually unveiled [17]. Accumulated studies have shown that exosomes derived from stem cells promote angiogenesis, inhibit ventricular remodeling, improve cardiac function, suppress local inflammation and regulate immunological response [18]. Exosomes derived from bone mesenchymal stem cells (BMSCs) ameliorated MI via inhibiting myocardial autophagy [19]. The placenta represents one of the most important sources of stem cells, in which high numbers of stem cells can be obtained by a non-invasive method [20]. Moreover, placental mesenchymal stromal cells (PMSCs) possess an angiogenic potential in cell therapy of MI [21]. However, the contribution of exosomes derived from human PMSCs (PMSC-Exos) in MI still remains largely unclear.

Taken together, we hypothesized that inflammation and alteration of gut microbiota may play a crucial role in the occurrence and development of MI. PMSC-Exos may represent a new approach for the treatment of MI via anti-inflammation and modulating gut microbiota. We firstly investigated the impacts and underlying mechanism of PMSC-Exos on the inflammation and gut microbiota in MI.

## Materials and methods

### Isolation and culture of human PMSCs

Human full-term placentas were obtained from healthy mothers at the time of routine elective cesarean section in General Hospital of Ningxia Medical University. Informed consent was obtained from each mother before the delivery. The Human Research Ethics Committee of Ningxia Medical University approved this study (No.2020-527). Human placental tissues were collected in accordance with a protocol approved by the Ethics Committee for the Conduct of Human Research of Ningxia Medical University. Fetal and maternal PMSCs were isolated and genetic origin verified as described previously [22, 23]. The cells were cultured in Dulbecco's modified eagle medium (DMEM, High glucose, Gibco, #12430104) supplemented with 10% fetal bovine serum (FBS, Gibco, #16000-044), 2 mM L-glutamine and 50 μg/mL gentamycin (Invitrogen, #R01510). This medium is referred to as PMSC medium. All cultures were maintained at 37 °C in a humidified incubator with 5% CO_2_. At about 90% confluence, the cells were passaged after detachment with TrypLE™ Express (Invitrogen). All the studies were performed with three passages of the established PMSCs culture. All methods were carried out in accordance with the 'Declaration of Helsinki'.

### Identification of human PMSCs by flow cytometry

Human PMSCs were identified by flow cytometry as previously described [22, 23]. PMSCs were harvested by TrypLE™ Express treatment and analyzed by flow cytometry with a FACS Calibur flow cytometer (BD Biosciences). All monoclonal antibodies used for flow cytometry were obtained from BD Biosciences and prepared in PBS. The final concentration of PMSCs was adjusted to 1 × 10^7^ cells/mL. Subsequently, 100 µL of suspended cells was stained with PE-conjugated CD73 antibody (#550257), APC-conjugated CD90 antibody (#559869), FITC-conjugated CD105 antibody (#561443), PC7-conjugated CD19 antibody (#557835) for 30 min. Similarly, another 100 µL of suspended cells was stained with PE-conjugated CD11b antibody (#555388), APC-conjugated CD34 antibody (#555824), FITC-conjugated CD45 antibody (#555482), and PC7-conjugated HLA-DR antibody (#560651) for 30 min. Meanwhile, the cells were stained with isotype-matched control antibodies, respectively. After washing, prepared samples were resuspended in 500 μL PBS and detected.

### Isolation and identification of exosomes derived from human PMSCs (PMSC-Exos)

PMSC-Exos were isolated with differential centrifugation as previously reported [24]. FBS was centrifuged using ultracentrifugation at 100,000 × *g* for 8 h to expel the PMSC-Exos. Once confluence of PMSCs reached 80%, the supernatant in the culture medium was dislodged and the suspension was washed 2 times with phosphate buffered saline (PBS, Hyclone, #SH30256.01B). The PMSCs were further incubated with 10% FBS culture solution without exosomes in a CO_2_ incubator at 37 °C for 48 h. The culture medium was centrifuged at 300 × *g* at 4 °C for 10 min. Furthermore, the supernatant was obtained and centrifuged 2 times at 2000 × *g* at 4 °C and 5000 × *g* at 4 °C for 15 min with the removal of the disposition. Then, the supernatant was finally centrifuged at 12,000 × *g* at 4 °C for 30 min, after which the disposition was collected and washed with PBS. Next, the suspension was centrifuged at 12,000 × *g* at 4 °C for 70 min for the collection of the disposition. After differential centrifugation, the supernatant was ultracentrifuged at 100,000 × *g* for 70 min with the collection of disposition. Lastly, the collected disposition was washed once using PBS and the cell suspension was centrifuged at 100,000 × *g* at 4 °C for 70 min to gather the disposition.

Exosomes were observed and identified by transmission electron microscope (TEM, Beijing SJC Science And Trade Co., Ltd., #HT7700 Exalens) [24, 25]. The prepared exosomes were fixed in 4% glutaric dialdehyde at 4 °C for 2 h, washed 3 times with PBS (0.1 mol/L), and later fixed with 1% osmium tetroxide for 2 h. Next, the exosomes were subjected to gradient dehydration with normal ethanol and acetone, followed by infusion, embedding, and polymerization with epoxy resin. The exosomes were finally sliced into 0.5 μm within sections for optical mirror positioning. Moreover, 60 nm ultrathin sections were prepared, stained with uranyl acetate and lead citrate, and further observed under a TEM. Moreover, we measured the particle sizes of PMSC-Exos with nanoparticle tracking analysis (NTA) using ZetaView PMX 110 (Particle Metrix) and its corresponding software ZetaView 8.04.02. PMSC-Exos were diluted in sterile PBS before NTA analysis [26]. NTA measurement was recorded and analyzed at 11 positions. The ZetaView system was calibrated using 110 nm polystyrene particles. The temperature was maintained at approximately 30℃.

### Animals and diet

Male C57BL/6 mice weighing 24–25 g were obtained from the Center for Experimental Animals of Ningxia Medical University. All animal experiments were approved by the Ethics Committee of Ningxia Medical University (No. 2016-232) and carried out in accordance with regulation of the Laboratory Animal Research Center of Ningxia Medical University. The animals were acclimatized to 1 week in polycarbonate cages at a temperature-controlled room (22 ± 2 °C, air humidity 40–70%) under a 12 h light and dark cycle. All animals were fed with commercial diet (46.65% crude protein, 20.73% moisture, 0.09% crude fat, 0.13% crude ash, 0.07% crude fiber, microelement calcium and phosphorus) from Keaoxieli Feed Co., Ltd.

### Establishment of MI model in mice and injection of exosomes

Mice were randomly divided into 3 groups (10 mice/group): Model group (injected with 200 mL PBS), PMSC-Exos group (injected with 200 mL PMSC-Exos), and Sham group (only performed thoracotomy without injection of any substance), establishment of mouse MI model as previously described [27]. Mice in the model and PMSC-Exos group were anesthetized with 1–5% isoflurane (Ning fen, #00021571) and oxygen at a flow rate of 2 L/min and kept in a surgical plane of anesthesia with 2% isoflurane during surgery. A left thoracotomy was carried out, and the heart was gently exposed from the pericardial sac through the incision. The LAD coronary artery was located and occluded with 6-0 polypropylene silk suture at about 2 mm from aortic root. The suture was tied, and the ligation was estimated to be successful when the anterior wall of the left ventricle turned pale. The heart was repositioned, then the chest was compressed to remove any air from the cavity, and the incision was closed using a purse string suture. Mice in sham-operated group that served as controls were subjected to similar surgical procedures except that the LAD coronary artery was not ligated. Mice were injected with their respective solution by tail intravenous on day 7, 9 and 11 after surgery. Body weights (BWs) were measured weekly. After 4 weeks, all mice were euthanized and associated indications were investigated.

### Electrocardiogram (ECG)

An EGG was recorded during the surgical period for 2 min before and 10 min after the coronary artery ligation in stabilized mice under isoflurane anesthesia by PowerLab data acquisition system (AD Instruments) [28]. Briefly, mice were placed on a small platform instrumented with ECG recording electrodes. All mice were allowed to acclimate for 10 min. After the acclimation period, ECG signals were recorded for 5 s while the mice passively established contact between the underside of their paws and the electrodes. All data was obtained during daylight hours, at which point the mouse heart rate was stable before increasing during the active evening/night hours.

### Assessment of cardiac function by cardiac echocardiography

An echocardiogram was obtained at 4 weeks post surgery by 2D guided M-mode and Doppler modalities with a 50-Hz probe (Vivid 7 Pro; GE Medical Systems) as described [27, 29]. 2D M-mode parasternal short-axis view images were obtained to determine systolic functional parameters such as LV end diastolic dimension (LVEDd), right ventricular end diastolic dimension (RVDd), LV ejection fraction (LVEF), LV fractional shortening(LVFS), LV end-diastolic volume (LVEDV), right ventricular end-diastolic volume (RVEDV) and LV posterior wall diastolic thickness (LVPWd). All echocardiographic images were analyzed to calculate the listed parameters using EchoPAC software (GE Medical Systems, Milwaukee, WI). All measurements were performed blinded and averaged from three cardiac cycles to account for inter-beat variability.

### Masson trichrome staining

Myocardial fibrosis was detected by Masson trichrome staining (Solarbio, #G1340) [30]. The heart tissues were fixed in 4% paraformaldehyde and cut into three thick slices from apex to base along the long axis using a tissue slicer. After embedded in paraffin and sectioned at 4 μm. Then sections were stained with masson trichrome with the following modification in the protocol. Briefly, myocardium sections were dehydrated by graded ethanol. Samples on slides were washed and stained with Weigert's iron hematoxylin solution (10 min), stained with acidic ethanol differentiation solution (10 s), rinsed in deionized water, stained with acid fuchsin (10 min), rinsed again and placed in phosphotungstic/phosphomolybdic acid solution (2 min), stained with aniline blue solution (2 min), placed in 1% acetic acid (1 min), rinsed and dehydrated through 70% and 90% ethanol, dimethylbenzene vitrification and deposited in holly oil. The collagen volume fraction in the infarcted and non infarcted areas of LV was calculated according to image. Myocardial fibrosis was expressed as a percentage of fibrotic area to left ventricle area (% of LV).

### Determination of plasma and myocardial biochemical indicators

Biochemical indications of lipid metabolism, including plasma total cholesterol (TC), high density lipoprotein cholesterol (HDL-C), as well as plasma and myocardial tissue aspartate transaminase (AST), were respectively determined using Hitachi 7180 automatic biochemical instrument (Olympus) [15]. The levels of plasma and myocardial tissue brain natriuretic factor or peptide (BNP, #F2060-B), myoglobin (MYO, #F2518-B), and troponin-I (Tn-I, #F2379-B) were measured by the commercial enzyme linked immunosorbent assay (ELISA) kit (Fankewei Biology) according to previous study [31]. In brief, firstly, samples or standards were added to the 96-well plates, followed by the antibody mix. After incubation, the wells were washed to remove unbound material. Tetramethylbenzidine (TMB) substrate was added, generating blue coloration. This reaction was then stopped by addition of stop solution completing any color change from blue to yellow. Signal was generated proportionally to the amount of bound analyte, and the intensity was measured at 450 nm. Each reaction was run in triplicate by the same operator.

### Determination of plasma and myocardial inflammatory factors

Myocardial tissues (100 mg) were homogenized by the Tissurelyser-24 Grinder. After centrifugation at 300 × *g* for 5 min, the supernatants of homogenates were collected for the determination of myocardial IL-1β, IL-6, and TNF-α concentrations. These plasma and myocardial inflammatory cytokines in each group were measured by Biolegend Enabling Legendary Discovery™ mouse inflammatory cytokine kits (Biolegend, #740150) [32]. The operation was performed thrice according to the manufacturer's instruction. In brief, after a series of cytokine standard dilutions were prepared, beads coated with 4 specific capture antibodies were mixed, respectively. Then 50 μL of the mixed captured beads, 50 μL of the plasma/myocardial supernatant samples or standard dilutions, and 50 μL of phycoerythrin (PE) detection reagent were added consecutively to each tube. All the tubes were gently mixed and incubated in the dark for 2 h at room temperature. The samples were washed with 1 mL of wash buffer and centrifuged at 1000 × *g* for 5 min. Then, all the tubes were added 25 μL of detection antibody and incubated the dark for 1 h at room temperature. Then, all the tubes were added 25 μL of SA-PE and incubated the dark for 30 min at room temperature. The samples were washed with 1 mL of wash buffer and centrifuged at 1000 × *g* for 5 min. The bead pellets were resuspended in 300 μL buffer after discarding the supernatant. The Beckman CytoFLEX FCM was sited with cytometer setup beads and the samples were measured. The data was analyzed using CytoFLEXFCM software (Beckman, USA). In addition, MCP-1 was measured by the commercial ELISA kit (Fankewei Biology, #F2082-A) as previously described [31].

### Gut microbiota sequencing analysis

According to previous detailed description [10], after 4 weeks of treatment, 5 mice from each group were randomly selected and placed in sterilized cages. The end of the tail and rectum of mice were squeezed gently. Then, feces were respectively collected with sterile tweezers into a sterile EP tube after defecation and immediately stored at − 80 °C for subsequent DNA extraction.

Microbial community genomic DNA was extracted from feces samples using the E.Z.N.A® soil DNA Kit (Omega Bio-Tek, #D3892-01) according to the manufacturer's instructions [15]. The DNA extract was checked on 1% agarose gel, and DNA concentration and purity were determined with NanoDrop 2000 UV–vis spectrophotometer (Thermo Scientific). The hypervariable region V3-V4 of the bacterial 16S rRNA gene was amplified with primer pairs 338F (5'-ACTCCTACGGGAGGCAGCAG-3') and 806R (5'-GGACTACHVGGGTWTCTAAT-3') by an ABI GeneAmp® 9700 PCR thermocycler (ABI). The PCR amplification of 16S rRNA gene was performed as follows: initial denaturation at 95 °C for 3 min, followed by 27 cycles of denaturing at 95 °C for 30 s, annealing at 55 °C for 30 s and extension at 72 °C for 45 s, and single extension at 72 °C for 10 min, and end at 4 °C. The PCR mixtures contain 5 × TransStart FastPfu buffer 4 μL, 2.5 mM dNTPs 2 μL, forward primer (5 μM) 0.8 μL, reverse primer (5 μM) 0.8 μL, TransStart FastPfu DNA Polymerase 0.4 μL, template DNA 10 ng, and finally ddH_2_O up to 20 μL. PCR reactions were performed in triplicate. The PCR product was extracted from 2% agarose gel and purified using the AxyPrep DNA Gel Extraction Kit (Axygen Biosciences, #AP-GX-500G) according to manufacturer's instructions and quantified using Quantus™ Fluorometer (Promega, #E615).

### Measurement of plasma LPS concentrations

Plasma LPS levels in each group were examined using limulus amebocyte lysate kit (Xiamen Bioendo Technology, #EC80545S) according to the manufacturer's instruction [33]. Briefly, 50 μL of diluted plasma (1:4 dilutions with endotoxin-free water) was dispensed to each well in a 96-well plate. At the initial time point, 50 μL of the limulus amebocyte lysate reagent was added respectively. The plate was incubated at 37 °C for 30 min. Then, 100 μL of chromogenic substrate warmed to 37 °C was added to each well, and incubation was extended for an additional 6 min at 37 °C. The reaction was stopped by adding 100 μL of 25% solution of glacial acetic acid. Optical density at 545 nm was measured with a microplate reader (Thermo Scientific, #20130199).

### Measurement of the feces SCFAs concentrations

Standard acetic acid, isobutyric acid, butyric acid, isovaleric acid and valeric acid at a minimum purity of 98% were obtained from Sigma-Aldrich (St. Louis). Phosphoric acid (#10015408) and ether (#10009318) with an analytical grade were purchased from Sinopharm Chemical Reagent Co., Ltd. SCFAs (acetic acid, isobutyric acid, butyric acid, isovaleric acid, and valeric acid) measurements were carried out on a single quadrupole mass spectrometer (#5975B-MSD) equipped with 6890N GC (Agilent Technologies) [15]. Samples were mixed with QL-866 vortex meter (Haimen) and separated from H1850R refrigerated centrifuge (Xiang Yi). A mixed standard stock solution to four SCFAs was prepared by dissolving an accurately weighed quantity of ether. Working solution series were prepared by appropriate dilutions of mixed standard stock solutions. Ten points calibration curve was made by adding the working solutions and an equal volume of IS solution covering a range from 0.05 to 250 μg/mL (0.05, 0.1, 0.5, 1, 5, 10, 25, 50, 100 and 250 μg/mL). All these solutions were stored in a freezer at 0 °C prior to use. Fecal samples weighing 100 mg were homogenized in 100 μL of 15% phosphoric acid with 100 μL of 250 μg/mL isocyanic acid solution as IS and 400 μL ether (70 Hz for 1 min). Subsequently, the samples were centrifuged at 4 °C for 10 min (12,000 × *g*) and the supernatants were transferred into the vial before gas chromatography mass spectrography (GC–MS) analysis. The GC was fitted with a capillary column Agilent HP-INNOWAX (30 m × 0.25 mm i.d. × 0.25 μL) (Agilent Technologies) and helium was used as the carrier gas at 1 mL/min. The injection was made in split mode at 10:1 with an injection volume of 1 μL and an injector temperature of 250 °C. The temperature of the ion source, interface, and quadrupole were 230 °C, 250 °C, and 250 °C, respectively. The column temperature was initially 90 °C, then increased to 120 °C at 10 °C/min, to 150 °C at 5 °C/min, and finally to 250 °C at 25 °C/min and kept at this temperature for 2 min (total run-time of 15 min). The detector was operated in electron impact ionization mode (electron energy 70 eV) using full scan and single ion monitoring (SIM) mode.

### Analysis of PMSC-Exos composition by whole transcriptome resequencing

In order to further reveal PMSC-Exos, the specific composition and content of possible mRNA and non-coding RNA (miRNA, lncRNA, cirRNA) in PMSC-Exos were analyzed by transcriptome high-throughput sequencing [34].

Firstly, RNA was extracted using the TRIzol method (TIANGEN BIOTECH) and treated with RNase-free DNase I (TaKaRa). RNA degradation and contamination was monitored on 1% agarose gels. RNA was quantified using Agilent 2100 Bioanalyzer (Agilent Technologies), the quality and integrity were assessed by NanoDrop spectrophotometer (IMPLEN).

Secondly, library preparation for small RNA sequencing: a total amount of 3 μg total RNA per sample was used as input material for the small RNA library. Sequencing libraries were generated using NEBNext® Multiplex Small RNA Library Prep Set for Illumina® (NEB) following manufacturer’s recommendations and index codes were added to attribute sequences to each sample. Briefly, NEB 3' SR Adaptor was directly, and specifically ligated to 3' end of miRNA, siRNA and piRNA. After the 3' ligation reaction, the SR RT Primer hybridized to the excess of 3' SR Adaptor (that remained free after the 3' ligation reaction) and transformed the single-stranded DNA adaptor into a double-stranded DNA molecule. This step is important to prevent adaptor-dimer formation. Besides, dsDNAs are not substrates for ligation mediated by T4 RNA Ligase 1 and therefore do not ligate to the 5 SR Adaptor in the subsequent ligation step. 5 ´ends adapter was ligated to 5 ´ends of miRNAs, siRNA and piRNA. Then the first strand cDNA was synthesized using M-MuLV Reverse Transcriptase (RNase H-). PCR amplification was performed using LongAmp Taq 2X Master Mix, SR Primer for illumina and index (X) primer. PCR products were purified on a 8% polyacrylamide gel (100 V, 80 min). DNA fragments corresponding to 140 ~ 160 bp (the length of small noncoding RNA plus the 3' and 5' adaptors) were recovered and dissolved in 8 μL elution buffer. Finally, library quality was assessed on the Agilent Bioanalyzer 2100 system using DNA High Sensitivity Chips. The library preparations were sequenced on an Illumina Hiseq 2500/2000 platform by the Beijing Allwegene Technology Company Limited and 50 bp single-end reads were generated.

Library preparation for lncRNA sequencing: A total amount of 3 µg RNA per sample was used as input material for the RNA sample preparations. Firstly, ribosomal RNA was removed by Epicentre Ribo-zeroTM rRNA Removal Kit (Epicentre, #MRZG12324), and rRNA free residue was cleaned up by ethanol precipitation. Subsequently, sequencing libraries were generated using the rRNA-depleted RNA by NEBNext® UltraTM Directional RNA Library Prep Kit for Illumina® (NEB) following manufacturer's recommendations. Briefly, fragmentation was carried out using divalent cations under elevated temperature in NEBNext First Strand Synthesis Reaction Buffer(5X). First strand cDNA was synthesized using random hexamer primer and M-MuLV Reverse Transcriptase(RNaseH-). Second strand cDNA synthesis was subsequently performed using DNA Polymerase I and RNase H. In the reaction buffer, dNTPs with dTTP were replaced by dUTP. Remaining overhangs were converted into blunt ends via exonuclease/polymerase activities. After adenylation of 3’ ends of DNA fragments, NEBNext Adaptor with hairpin loop structure was ligated to prepare for hybridization. In order to select cDNA fragments of preferentially 150–200 bp in length, the library fragments were purified with AMPure XP system (Beckman Coulter, Beverly, USA). Then 3 ul USER Enzyme (NEB) was used with size-selected, adaptor-ligated cDNA at 37 ℃ for 15 min followed by 5 min at 95 ℃ before PCR. Then PCR was performed with Phusion High-Fidelity DNA polymerase, Universal PCR primers and Index (X) Primer. Finally, products were purified (AMPure XP system) and library quality was assessed on the Agilent Bioanalyzer 2100 system. The library preparations were sequenced on an Illumina Hiseq 4000 platform by Beijing Allwegene Technology Company Limited and paired-end 150 bp reads were generated.

Thirdly, raw data (raw reads) of fastq format were firstly processed through in-house perl scripts. In this step, clean data (clean reads) were obtained by removing reads containing adapters, reads containing ploy-N and low quality reads from raw data. At the same time, Q20, Q30 and GC content of the clean data were calculated. All the downstream analyses were based on clean data with high quality.

Finally, Gene Ontology (GO) enrichment analysis of differentially expressed genes or lncRNA target genes were implemented by the GOseq R package, in which gene length bias was corrected. GO terms with corrected p value less than 0.05 were considered significantly enriched by different expressed genes. Kyoto Encyclopedia of Genes and Genomes (KEGG) is a database resource for understanding high-level functions and utilities of the biological system, such as the cell, the organism and the ecosystem, from molecular-level information, especially large-scale molecular datasets generated by genome sequencing and other high-throughput experimental technologies. We used KOBAS software to test the statistical enrichment of differential expression genes or lncRNA target genes in KEGG pathways.

### Statistical analysis

Statistical analysis was performed using GraphPad Prism software 6.01 (GraphPad Software Inc., CA, USA) and SPSS 17.0 (IBM Corp., NY, USA). Results were expressed as the mean ± standard deviation (SD). Statistical significance was evaluated by one-way analysis of variance (ANOVA) followed by Tukey Kramer multiple comparison test or Student’s t-test. Moreover, Spearman's correlation analysis was performed to identify the correlations between microbiota and inflammatory indicators. *P* < 0.05 was considered to be statistically significant.

## Results

### Identification of PMSCs and PMSC-Exos

**Fig.1:**
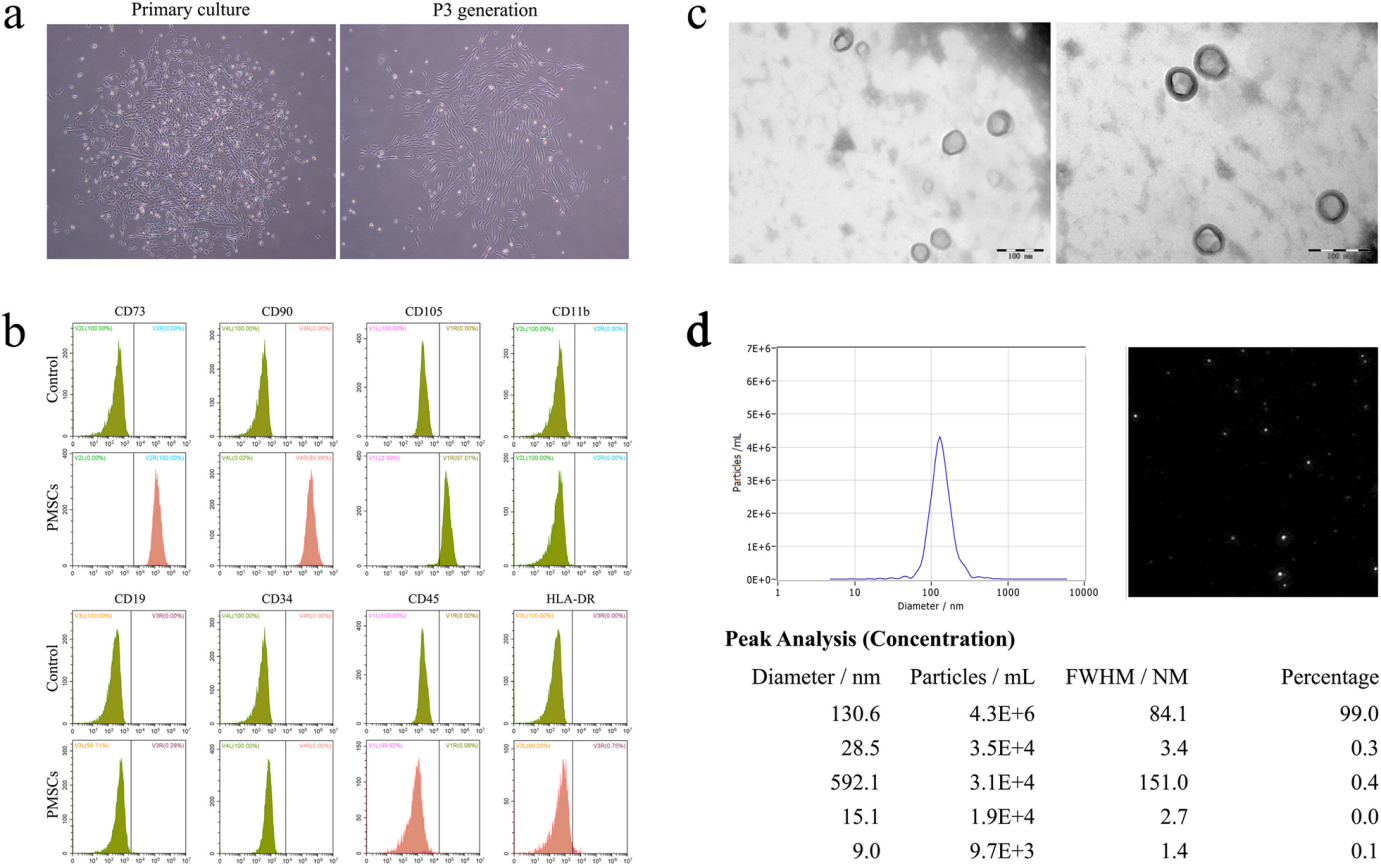
Morphological characteristics and identification of PMSCs and exosomes. **a** Morphological characteristics of PMSCs. **b** Positive rates of cell surface antigens CD73, CD90, CD105, CD11b, CD19, CD34, CD45 and HLA-DR in PMSCs were detected by flow cytometry. **c** Observation of exosomes by transmission electron microscopy (TEM). **d** Observation of exosomes by nanoparticle tracking analysis (NTA)

PMSCs isolated and inoculated for the first time showed adherent growth and vortex growth. After 24 h, only a few cells were able to grow adhering to the wall. On the 6-7th day after inoculation, PMSCs were presented as fusiform with uniform shape and formed large cell clonal clusters (Fig. 1a). After 3 times of passage, the morphology of PMSCs was mainly stable spindle. (Fig. 1a). The positive rates of cell surface antigens including CD73, CD90, CD105, CD11b-PE, CD19-PC7, CD34-APC, CD45-FITC and HLA-DR-PC7 were 100.00%, 99.98%, 97.01%, 0.00%, 0.29%, 0.00%, 0.08% and 0.75%, respectively (Fig. 1b), indicating the primary cultured PMSCs with a high purity.

To confirm the successful extraction of PMSC-Exos, we characterized them using TEM. Observations of the PMSC-Exos revealed specific round or elliptical shapes, with a diameter of about 60–200 nm, a complete membrane structure and low-density substances inside (Fig. 1c). Moreover, NTA indicated that the main peak size of exosomes centered around approximately 60–200 nm (Fig. 1d). Exosomes with the size of 130.6 nm accounted for 99% of the total, suggesting successful isolations of human PMSC-Exos.

### Identification of mice model of MI by EGG

**Fig. 2:**
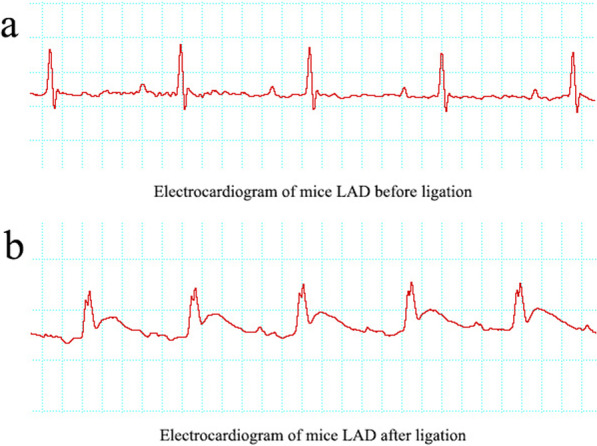
Identification of myocardial infarction in mice. **a** Electrocardiogram of mice left anterior descending coronary artery (LAD) before ligation. **b** Electrocardiogram of mice LAD after ligation

To assess the role of PMSC-Exos on MI, firstly, a classical mouse MI model was generated in this study according to previous descriptions [19]. A total of 30 mice were used to establish the model of MI and 24 mice eventually survived. The EGG II was observed with normal P wave and QRS wave before the ligation of mouse LAD coronary artery (Fig. 2a). Intriguingly, the myocardium below the ligation site was pale immediately and the beat was significantly weakened after the ligation. Furthermore, the amplitude of the QRS wave became larger and ST wave fusion was raised, indicating that ischemic necrosis of the myocardium layer and MI model were successfully established (Fig. 2b).

### PMSC-Exos improved cardiac function and reduced MI area

**Fig. 3:**
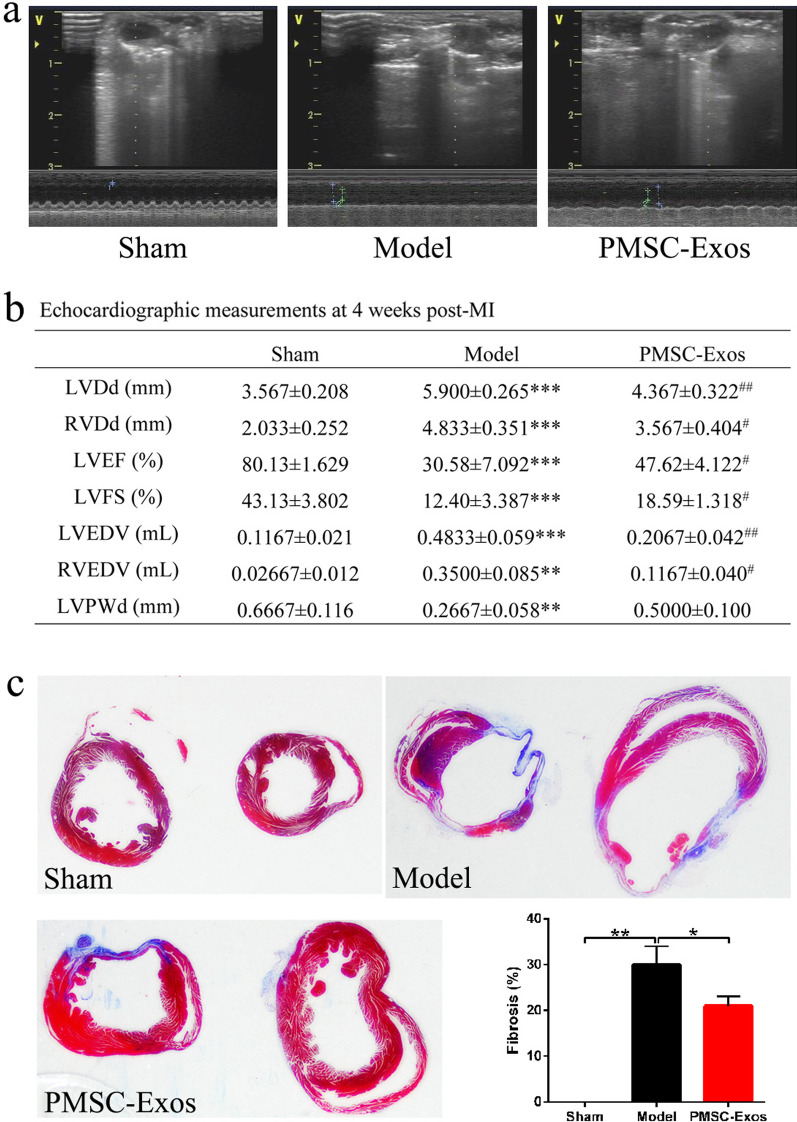
PMSCs-derived exosomes improved cardiac function and myocardial infarction (MI) area of mice with MI**. a** Representative images of echocardiograms in diverse groups. **b** Determination of LVDd, RVDd, LVEF, LVFS, LVEDV, RVEDV and LVPWd of mice in each group by echocardiography. Data were expressed as mean ± SD. **P < 0.01, ***P < 0.001 versus sham. ^#^*P* < 0.05, ^##^*P* < 0.01 versus model. **c** Exosomes secreted by PMSCs reduced myocardial infarction area of mice with MI. Myocardial tissue was stained in red and collagen fibers were stained in blue by Masson staining. Data were expressed as mean ± SD. *P < 0.05, **P < 0.01

Representative images of the M-mode view of cardiac performance in diverse groups demonstrated that compared with the sham group, LVDd (*P* = 0.0003), RVDd (*P* = 0.0004), LVEDV *(P* = 0.0005), RVEDV (*P* = 0.0029) and LVPWd (*P* = 0.0058) in the model group were significantly increased, implying LV dilatation (Fig. 3a, b). After PMSCs-Exos injection, LVDd (*P* = 0.0031), RVDd (*P* = 0.0149), LVEDV *P* = 0.0026) and RVEDV (*P* = 0.0129) were decreased in comparison with sham group (Fig. 3b). Additionally, the systolic function was reduced in the MI group as evidenced by significantly decreased values of LVEF (*P* = 0.0003) and LVFS (*P* = 0.0005), compared to the sham group (Fig. 3b). Importantly, PMSCs-Exos treatment elevated LVEF (*P* = 0.0228) and LVFS (*P* = 0.042) (Fig. 3b).

Estimation of myocardial fibrosis suggested a significant collagen accumulation in model group (*P* = 0.002, Fig. 3c). Conversely, PMSCs-Exos administration significantly decreased the fibrosis area in comparison with model group (*P* = 0.0252), but still showed higher than sham group (Fig. 3c).

### PMSC-Exos reduced characteristic indicators in MI

**Fig. 4:**
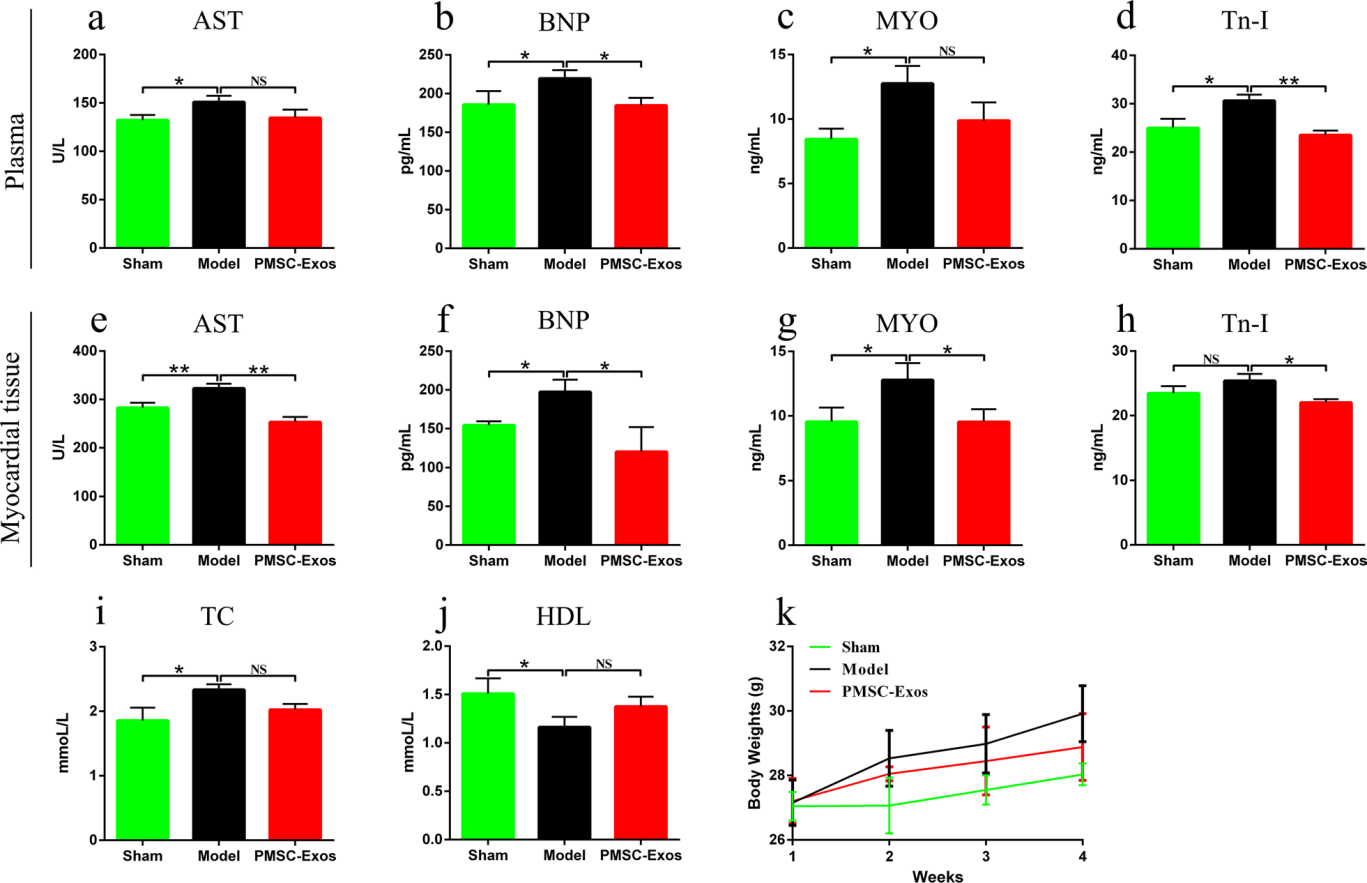
PMSCs-derived exosomes reduced plasma and myocardial biochemical indicators. **a** Plasma aspartate transaminase (AST) levels. **b** Plasma brain natriuretic peptide (BNP) levels. **c** Plasma myoglobin (MYO) levels. **d** Plasma troponin- I (Tn-I) levels. **e** Myocardial AST levels. **f** Myocardial BNP levels. **g** Myocardial MYO levels. **h** Myocardial Tn-I levels. **i** Plasma total cholesterol (TC) levels. **j** Plasma high density lipoprotein cholesterol (HDL-C) levels. **k** BWs growth curve. Data were expressed as mean ± SD. *P < 0.05, **P < 0.01, NS: *P* > 0.05

To further estimate the amelioration of PMSC-Exos on MI, the characteristic indicators including AST, BNP, MYO, Tn-I from plasma or myocardial, as well as plasma TC, HDL levels were investigated, respectively (Fig. 4a–k). The results showed that levels of plasma AST (*P* = 0.0182), BNP *P* = 0.0469), MYO (*P* = 0.092), Tn-I (*P* = 0.0129) and TC (*P* = 0.0193) in model group were increased, compared to sham group (Fig. 4a–d, i). PMSCs-Exos treatment decreased plasma BNP (*P* = 0.0149) and Tn-I (*P* = 0.0014) compared to model group (Fig. 4b, d). Similarly, myocardial AST (*P* = 0.0081), BNP (*P* = 0.0111) and MYO (*P* = 0.0305) in model group were higher than sham group, whereas PMSCs-Exos treatment reduced the levels of AST (*P* = 0.0011), BNP (*P* = 0.0199), MYO (*P* = 0.0265) and Tn-I (*P* = 0.0184) in comparison with model group (Fig. 4e–h). Moreover, plasma HDL level in model group was lower than that in sham group (*P* = 0.0337), which could not be attenuated by PMSCs-Exos administration **(P** > 0.05, Fig. 4j). Additionally, there was no significant difference in initial BWs or terminal BWs among diverse groups (*P* > 0.05, Fig. 4k).

### PMSC-Exos ameliorated plasma and myocardial inflammatory indicators

**Fig. 5:**
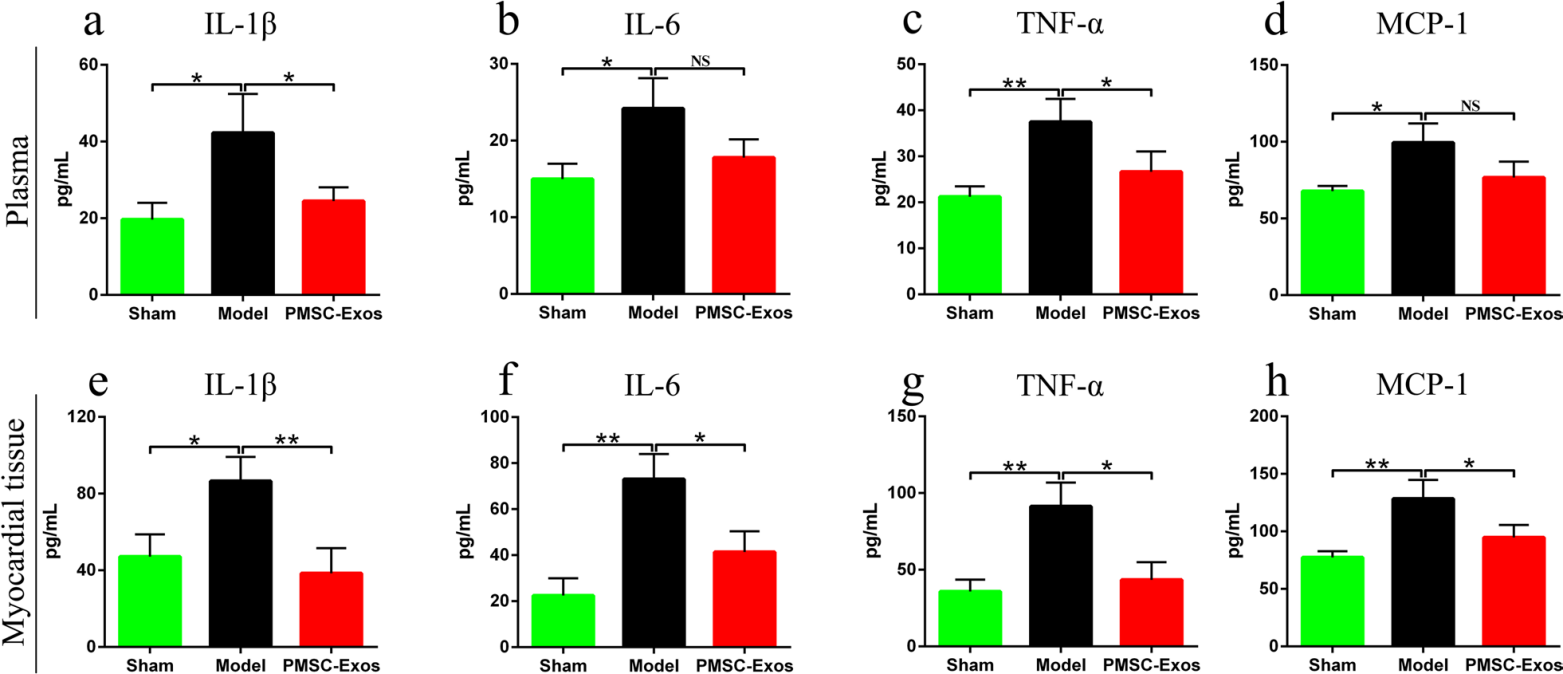
PMSCs-derived exosomes ameliorated plasma and myocardial inflammation factors. **a** Plasma interleukin 1β (IL-1β) levels. **b** Plasma IL-6 levels. **c** Plasma tumor necrosis factor α (TNF-α) levels. **d** Plasma monocyte chemotactic protein 1 (MCP-1) levels. **e** Myocardial IL-1β levels. **f** Myocardial tissue IL-6 levels. **g** Myocardial TNF-α levels. **h** Myocardial MCP-1 levels. Data were expressed as mean ± SD. *P < 0.05, **P < 0.01, NS: *P* > 0.05

Due to a critical role of inflammation injury in the pathogenesis of MI, the levels of inflammatory indicators in plasma and myocardial tissue of diverse groups were respectively measured (Fig. 5a–h). Elevated plasma levels of pro-inflammatory IL-1β (*P* = 0.0241), IL-6 (*P* = 0.0229), TNF-α (*P* = 0.0068) and MCP-1 (*P* = 0.0133) were found in the model group, compared to sham group (Fig. 5a–d). However, these elevated IL-1β (*P* = 0.0462) and TNF-α (*P* = 0.0483) in plasma were reversely suppressed by PMSCs-Exos administration (Fig. 5a, c). Consistently, myocardial IL-1β (*P* = 0.0146), IL-6 (*P* = 0.0027), TNF-α (*P* = 0.0051) and MCP-1 (*P* = 0.0069) levels in model group were notably higher than those in sham group (Fig. 5e–h). Whereas, PMSCs-Exos treatment attenuated the myocardial in situ inflammation by decreasing IL-1β (*P* = 0.009), IL-6 (*P* = 0.0181), TNF-α (*P* = 0.0126) and MCP-1 (*P* = 0.0415) in MI (Fig. 5e–h). Taken together, these results demonstrated that PMSC-Exos intervention exhibited an anti-inflammation capacity in MI.

### PMSC-Exos altered the compositions of gut microbiota in MI

**Fig. 6:**
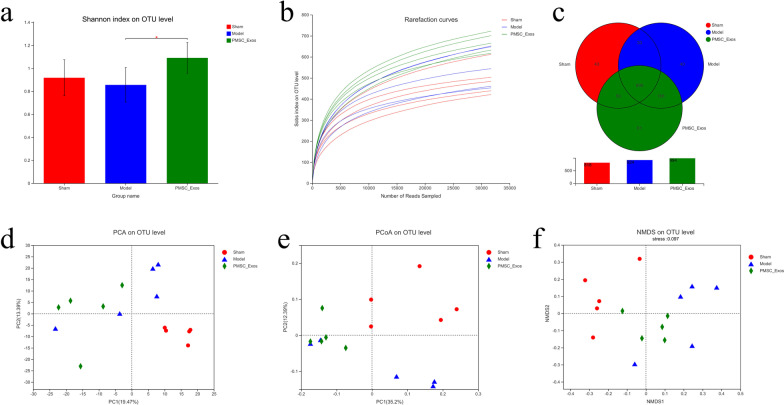
Alpha-diversity and β diversity of gut microbial in the feces of mice. **a** Shannon index on OTU level. **b** Rarefaction curves (Alpha-diversity analysis). **c** OUT analysis. **d** PCA analysis on OTU level. **e** PCoA analysis on OTU level. **f** NMDS analysis on OTU level

Numerous studies have increasingly demonstrated that the contribution of gut dysbiosis to the pathogenesis of MI [9, 35]. To assess whether PMSC-Exos effectiveness were correlated to gut microbial community in MI, the fecal samples of mice in diverse groups were measured by 16S rRNA high throughput sequencing and analysis. As shown in shannon index based on operational taxonomic unit (OTU) level, PMSC-Exos markedly increased gut bacterial diversity in mice (*P* < 0.05) in comparison with model (Fig. 6a). Rarefaction curves tended to be flat when the number of sequence increased to 20,000, indicating that the 16S rRNA sequencing were reasonable (Fig. 6b). As shown in venn diagram (Fig. 6c), there were 686 species shared among sham, model and PMSC-Exos groups by OUT analysis, whereas differential specific species in diverse groups including 43 species in sham group, 40 species in MI group as well as 81 OTUs in PMSC-Exos group. The diversity of gut microbiota was weak in MI group compared to sham group, which was positively modulated by PMSC-Exos administration. Moreover, principal component analysis (PCA) showed that there was an obvious species difference in diverse groups (Fig. 6d), indicating that PMSCs-Exos treatment may notably alter the overall composition of gut microbiota in MI. In parallel with PCA, principal co-ordinates analysis (PCoA) and non-metric multi-dimensional scaling (NMDS) analyses showed similar results (Fig. 6e, f).

**Fig. 7:**
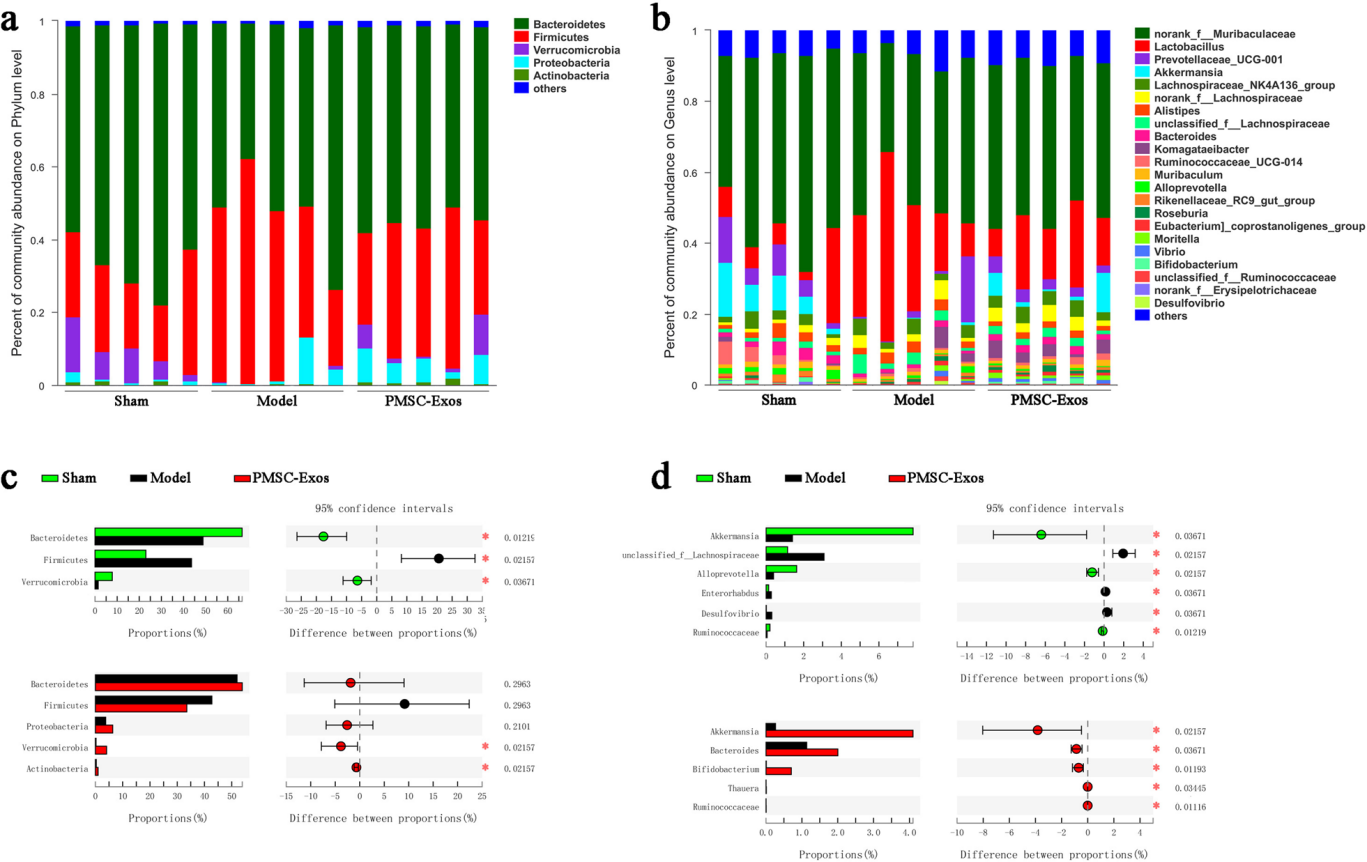
Relative abundances of gut microbial species at the phylum and genus levels in the feces of mice. **a**, **c** The relative abundances of gut microbial species in phylum level. **b**, **d** The relative abundances of microbial species in genus level. Data were expressed as mean ± SD. *P < 0.05

At the phylum level, *Bacteroidetes* and *Firmicutes* were predominant in diverse groups (Fig. 7a). The proportion of *Firmicutes* (*P* = 0.0216) in model group was higher than that in sham group. In contrast, *Bacteroidetes* (*P* = 0.0122) and *Verrucomicrobia* (*P* = 0.0367) in model group were lower than those in sham group (Fig. 7c). Moreover, the relative abundances of *Verrucomicrobia* (*P* = 0.0216) and *Actinobacteria* (*P* = 0.0216) in PMSC-Exos group were increased in comparison with model group (Fig. 7c). However, *Bacteroidetes*, *Firmicutes* and *Proteobacteria* showed no difference between model group and PMSC-Exos group (Fig. 7c).

At the genus level (Fig. 7b, d), the relative abundances of *Akkermansia* (*P* = 0.0367), *Alloprevotella* (*P* = 0.0216) and *Ruminococcaceae* (*P* = 0.0122) in MI group were markedly reduced, whereas the proportions of *unclassified_Lachnospiraceae* (*P* = 0.0216), *Enterorhabdus* (*P* = 0.0367) and *Desulfovibrio* (*P* = 0.0367) in MI group were obviously elevated, compared to sham group (Fig. 7d). Importantly, the proportions of *Akkermansia* (*P* = 0.0216), *Bacteroides* (*P* = 0.0367), *Bifidobacterium* (*P* = 0.0120), *Thauera* (*P* = 0.0345) and *Ruminococcaceae* (*P* = 0.0111) in PMSC-Exos group were higher than those in model group (Fig. 7d). Taken together, our data revealed that under this experimental condition, PMSC-Exos supplementation had a major effect on MI abnormal *Verrucomicrobia*, *Akkermansia* and *Ruminococcaceae*.

### PMSC-Exos reduced plasma LPS levels

**Fig. 8:**
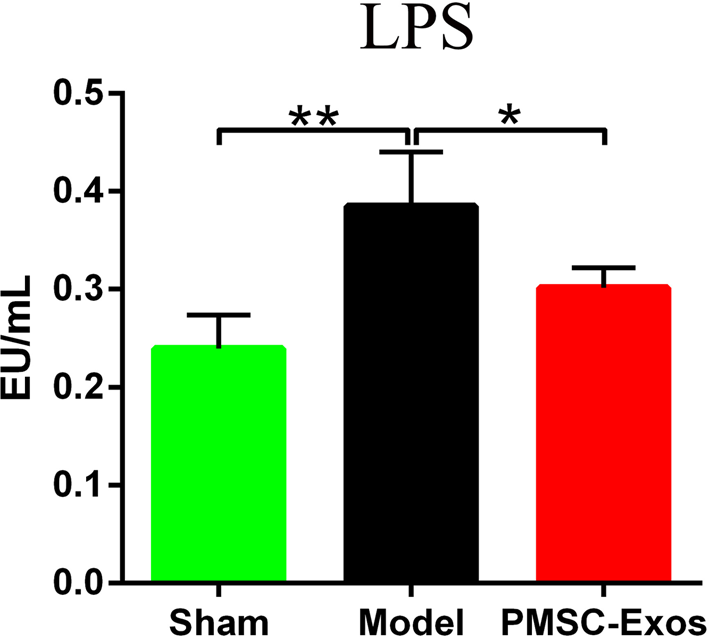
PMSC-Exos reduced plasma LPS levels. Data were expressed as mean ± SD. *P < 0.05, **P < 0.01

To further assess the effects of PMSC-Exos on intestinal permeability and integrality of MI after the above rectification of gut dysbiosis, circulating translocated LPS derived from Gram-negative bacteria was detected by limulus reagent. Plasma LPS concentration in model group was significantly higher than that in sham group (*P* = 0.0042), whereas PMSC-Exos reduced LPS in MI (*P* = 0.0299), suggesting that supplementary PMSC-Exos attenuated the endotoximia in MI (Fig. 8).

### PMSC-Exos modulated SCFAs contents of gut microbiota

**Fig. 9:**
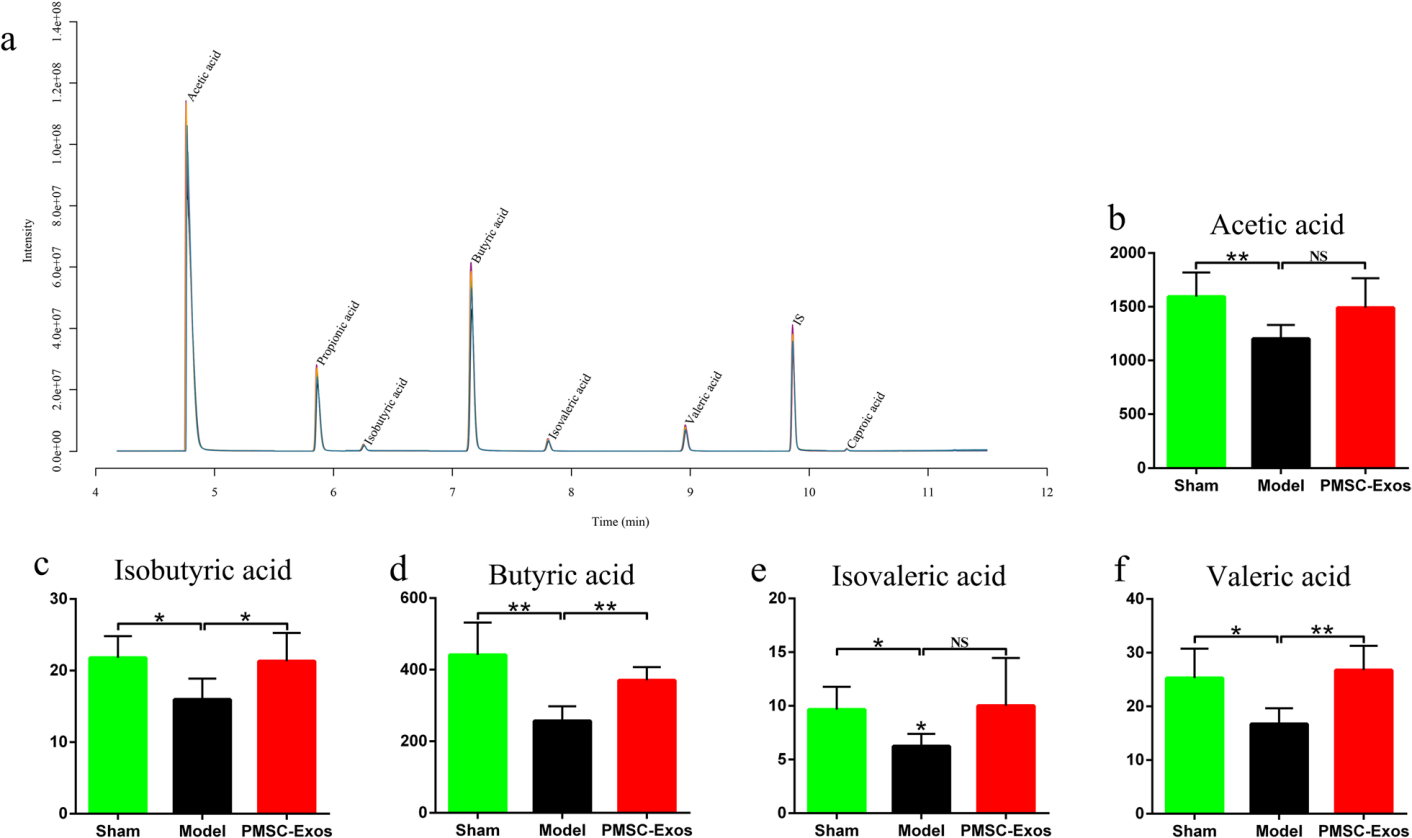
Effects of exosomes secreted by PMSCs on short-chain fatty acids (SCFAs) of diverse groups. **a** Sample chromatogram of rats stool. **b** Acetic acid. **c** Isobutyric acid. **d** Butyric acid. **e** Isovaleric acid. **f** Valeric acid. Data were expressed as mean ± SD. *P < 0.05, **P < 0.01, NS: *P* > 0.05

SCFAs mainly including acetic acid, isobutyric acid, butyric acid, isovaleric acid and valeric acid, represent critical metabolites of gut microbiota. As a result of determination by GC–MS (Fig. 9a), acetic acid (*P* = 0.0097), isobutyric acid (*P* = 0.0145), butyric acid (*P* = 0.0032), isovaleric acid (*P* = 0.0133) as well as valeric acid (*P* = 0.0146) in model group were significantly decreased compared with sham group (Fig. 9b–f). Intriguingly, after PMSC-Exos administration, the levels of isobutyric acid (*P* = 0.0405), butyric acid (*P* = 0.0018) and valeric acid (*P* = 0.0032) were obviously increased in comparison with model group (Fig. 9c, d, f). There’s no significant difference in acetic acid or isovaleric acid between model group and PMSC-Exos group (*P* > 0.05, Fig. 9b, e).

### Correlation analysis

**Fig. 10:**
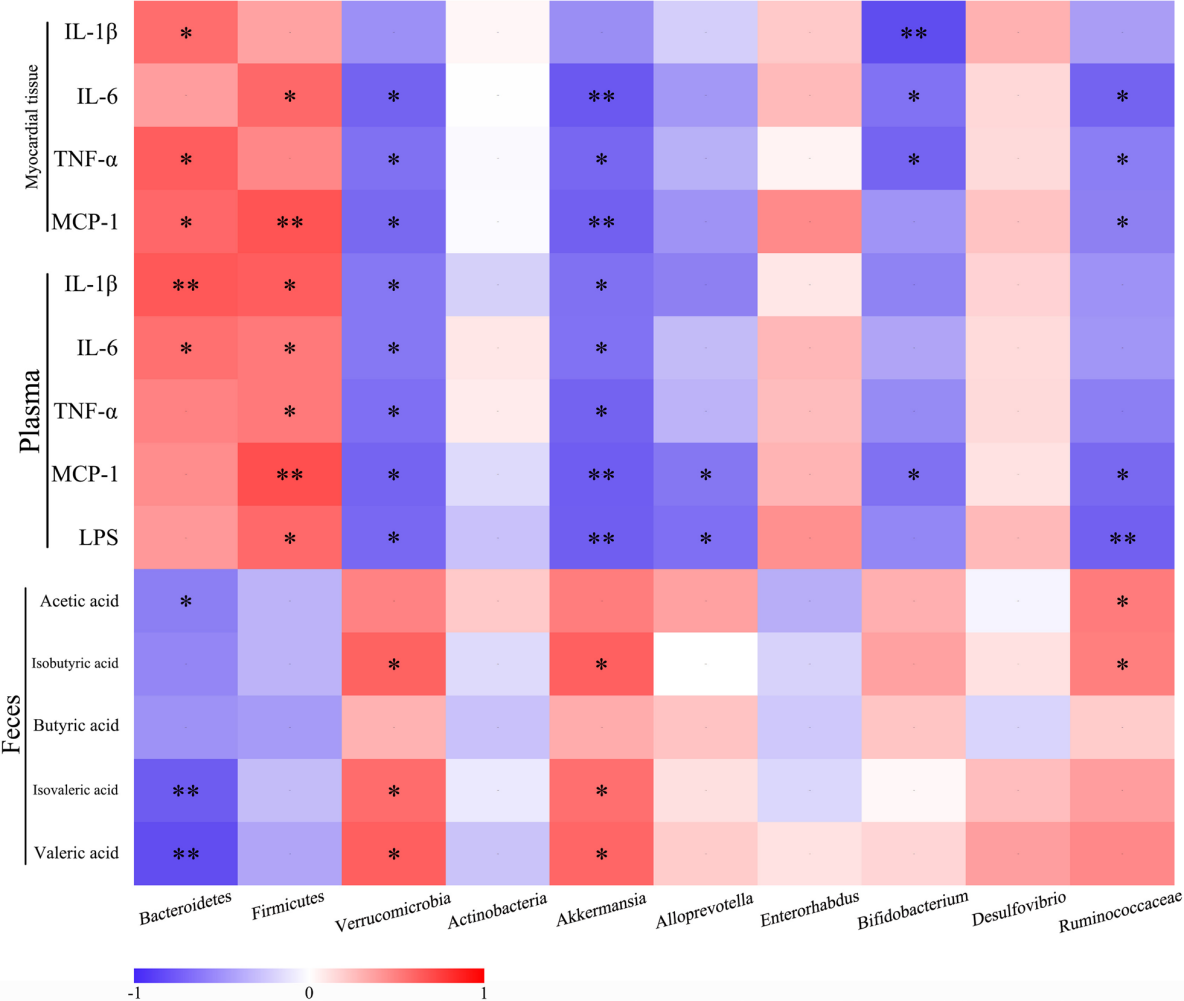
Correlation analyses between relative abundance (%) of microbiota and inflammation indicators. *P < 0.05, **P < 0.01

Correlations were separately analyzed among inflammatory indicators, differential bacteria and SCFAs (Fig. 10). The relative abundance of *Bacteroidetes* was negatively correlated with pro-inflammatory myocardial indicators (IL-1β, TNF-α and MCP-1) and plasma indicators (IL-1β and IL-6), whereas positively correlated with acetic acid, isovaleric acid and valeric acid, respectively. Similarly, *Firmicutes* was positively correlated with the levels of myocardial IL-1β and MCP-1, as well as plasma IL-1β, IL-6, TNF-α, MCP-1 and LPS. In contrast, *Verrucomicrobia* and *Akkermansia* were negatively correlated with myocardial indicators (IL-6, TNF-α and MCP-1) and plasma indicators (IL-1β, IL-6, TNF-α, MCP-1 and LPS), whereas positively correlated with isobutyric acid, isovaleric acid and valeric acid, respectively. Moreover, *Alloprevotella* was negatively correlated with plasma MCP-1 and LPS, respectively. Additionally, the proportion of *Bifidobacterium* was negatively correlated with inflammatory myocardial indicators (IL-1β, IL-6 and TNF-α) and plasma MCP-1, respectively. Similarly, the proportion of *Ruminococcaceae* was negatively correlated with myocardial indicators (IL-6, TNF-α and MCP-1) and plasma indicators (MCP-1 and LPS), whereas positively correlated with acetic acid and butyric acid, respectively. These results indicated that gut microbiota and inflammatory indicators interfered and closely correlated in MI.

### Whole transcriptome resequencing analysis of PMSC-Exos

**Fig. 11:**
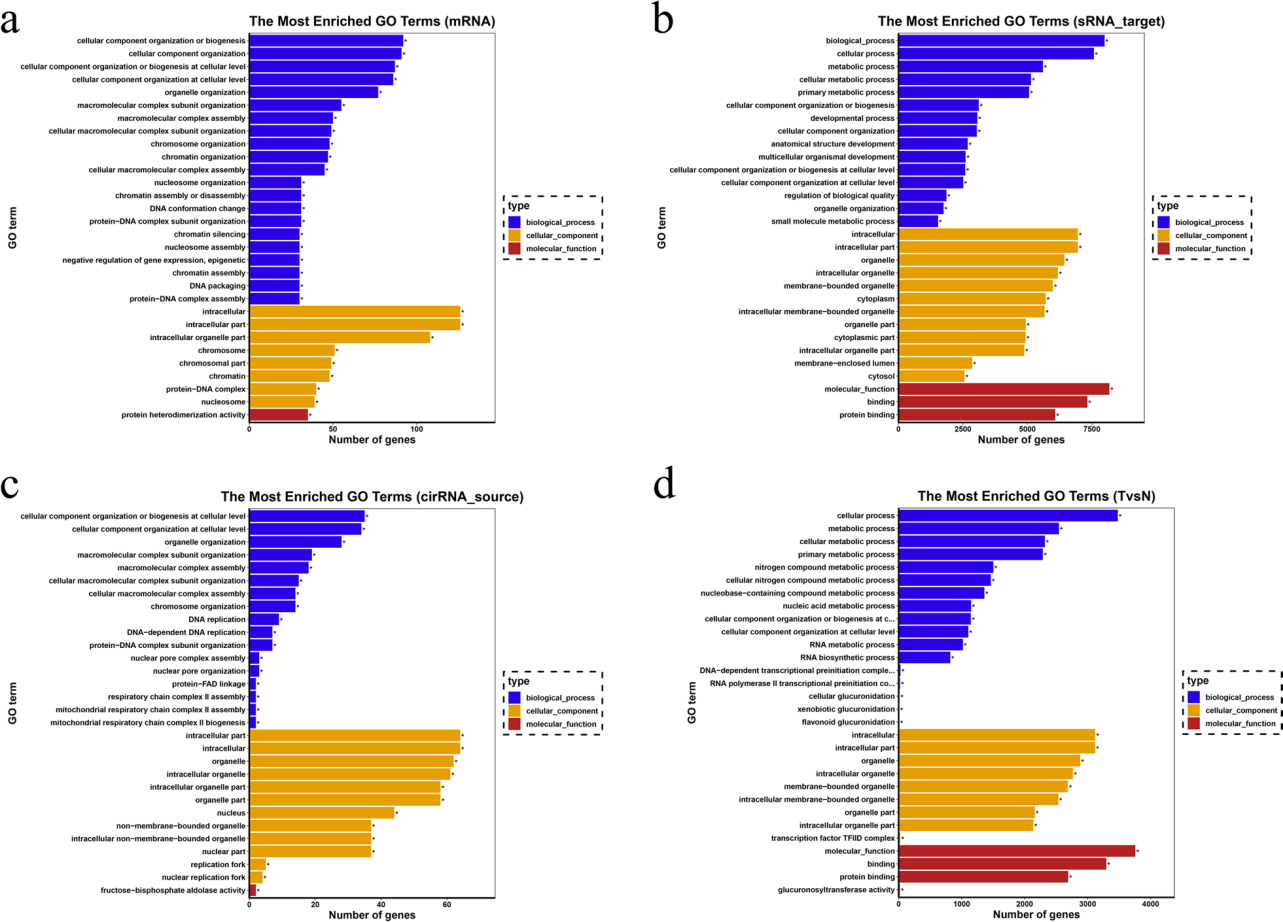
GO analysis. **a** mRNA. **b** sRNA. **c** cirRNA. **d** TvsN

**Fig. 12:**
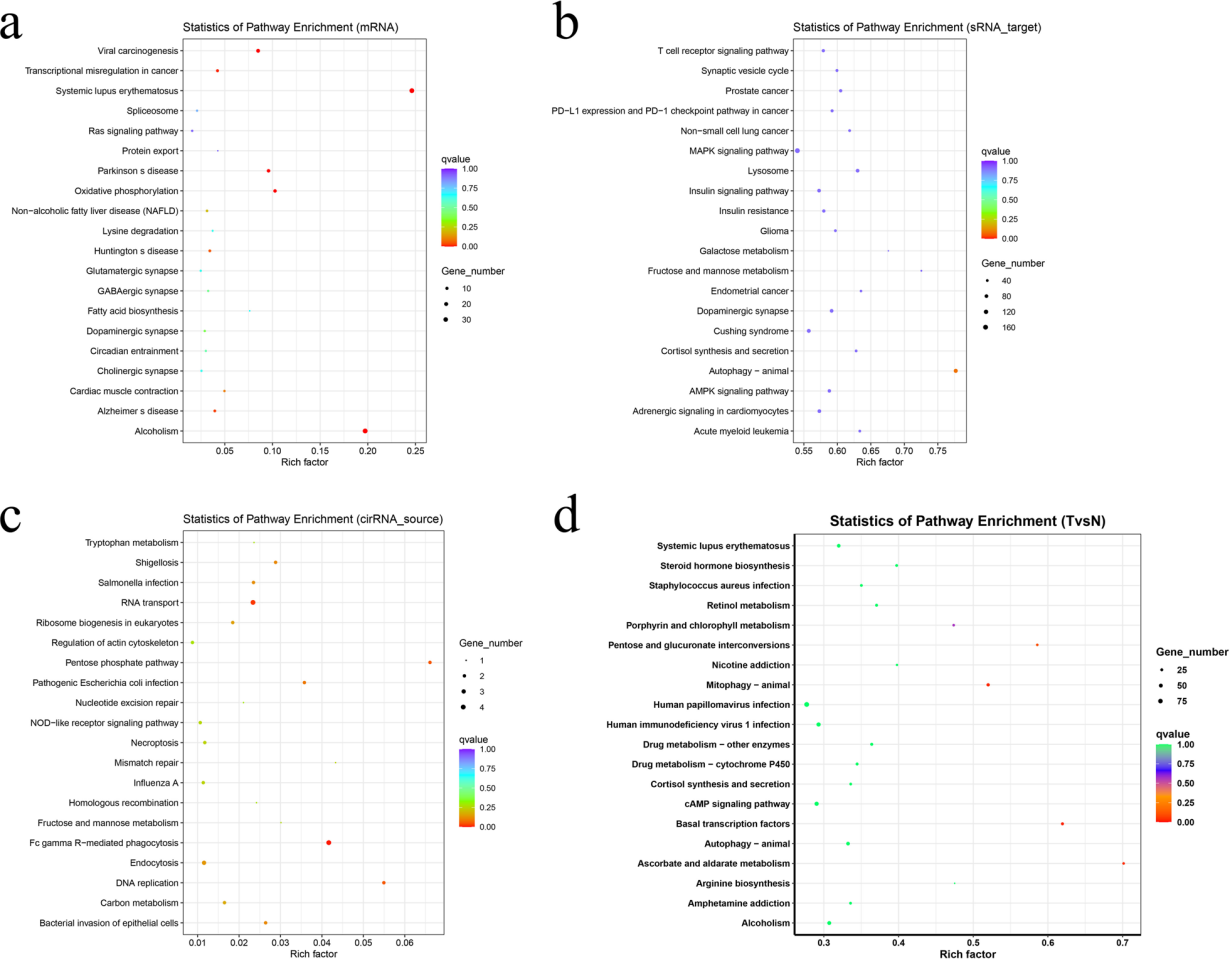
KEGG functional enrichment analysis. **a** mRNA. **b** sRNA. **c** cirRNA. **d** TvsN. KEGG pathway were analyzed using the KEGG database of the *Kanehisa* laboratory^37^–^39^ (www.kegg.jp/kegg/kegg1.html)

Exosomes have been thought to be critical carriers of biological information for facilitating intercellular communication [36]. To determine the contents in PMSC-Exos, high throughput transcriptome sequencing was performed. After bioinformatics analysis by FastQC software, the quality value of the sequencing sample Q20 was 99.37–99.41%, and Q30 was 97.93–98.03%, meeting the criteria for subsequent data analysis (Table 1). GO analysis (mRNA, miRNA, cirRNA, lncRNA) showed predominant changes were involved in the biological process including cellular component organization or biogenesis, cellular component organization, cellular process, cellular component organization or biogenesis at cellular level and cellular component organization at cellular level, in cellular component belonging to intracellular, intracellular part and organelle, in molecular function with protein heterodimerization activity, binding, protein binding, fructose-bisphosphate aldolase activity and glucuronosyl transferase activity) (Fig. 12a–d). Collectively, these results demonstrated that PMSC-Exos possessed multi-functional contents at transcriptional level, which may probably contribute to the potential effects on MI (Fig. 11a–d).

**Table 1:** Quality control

Sample	Reads	Bases	Error rate (%)	Q20 (%)	Q30 (%)	GC content (%)
1	13,619,873	0.68G	0.01	99.37	97.93	56.60
2	18,721,563	0.93G	0.01	99.41	98.03	59.28

*Sample* sample ID, *Reads* the number of sequencing sequences of each sequencing file is counted in a unit of four sequences, *Bases* the number of sequenced sequences is multiplied by the length of sequenced sequences and converted to units of G, *Error rate* the sequencing error rate was calculated by Formula 1, *Q20* the percentage of bases with Phred greater than 20 in the population, *Q30* the percentage of bases with Phred greater than 30 in the population, *GC content* calculate the total number of bases G and C as a percentage of the total number of bases

KEGG pathway were analyzed using the KEGG database of the *Kanehisa* laboratory [37–39]. KEGG functional enrichment analysis (mRNA, miRNA, cirRNA, lncRNA) showed the remarkable alterations were embodied in the systemic lupus erythematosus, alcoholism, autophagy-animal, pentose phosphate pathway, DNA replication, Fc gamma R-mediated phagocytosis, pathogenic *Escherichia coli* infection, ascorbate and alternate metabolism, basal transcription factors, pentose and glucuronate interconversions and mitophagy-animal (Fig. 12).

## Discussion

Our study originally highlighted that PMSC-Exos played an important role in alleviation of cardiac function and myocardial infarct area in MI mice. Moreover, the effectiveness of PMSCs-Exo on MI may be closely associated with the suppression of inflammation and the rectification of gut dysbiosis. This study may potentially promote the understanding of underlying mechanism in MI and provide a novel therapeutic PMSC-Exos for the control of MI.

In our study, the primary cultured human PMSCs were positive for predominant markers (CD73, CD90, and CD105), which was consistent with previous study [40], indicating obtained PMSCs with high purity and uniformity. Subsequently, TEM and NTA results revealed that PMSC-Exos possess the characteristics of exosomes and accounted for 99% of the total, which was parallel with previous studies [41], suggesting successful isolation of human PMSC-Exos. In the present study, we hypothesized that PMSC-Exos may represent a novel alternative treatment in MI.

After LAD coronary artery ligation, abnormal EGG, increased myocardial fibrosis and LV dilatation demonstrated that MI models were successfully established, which was consistent with previous description [2]. A recent research supported that exosomes play a particularly prominent role in the improvement of infarction area as well as heart function in MI [18]. Similarly, in our study, exosomes derived from PMSC obviously alleviated LV dilatation and fibrosis area, indicating that PMSC-Exos were capable of improving myocardial function in MI.

Dyslipidemia is a key factor in the development of MI and its subsequent complications [42]. Clinical studies have found that increased TC and LDL-C, as well as decreased HDL-C were associated with elevated risk of MI, which was also appeared in our MI model [43]. Moreover, AST, Tn-I, MYO and BNP are sensitive markers of myocardial cell injury or death after MI [44, 45]. A survey has suggested that MYO, Tn-I, and plasma NT-proBNP levels together can significantly contribute to the early diagnosis of AMI [46]. In our study, PMSC-Exos administration reduced abnormally elevated cardiac biomarkers including AST, BNP, MYO and Tn-I, demonstrating PMSC-Exos could improve myocardial cell injury/death in MI.

To further investigate the underlying mechanisms of the effectiveness of PMSC-Exos in MI, firstly, the dynamic alterations of inflammation were determined in diverse groups. Accelerating studies have indicated that inflammation plays a vital role in the process in MI by the release of cytokines from damaged myocardium and the activation of severe inflammatory cascade reaction [5, 47, 48]. In the first 24–72 h after the MI, the impairment of cardiomyocytes triggered damage-associated molecular patterns (DAMPs)-mediated activation of macrophages to secret pro-inflammatory cytokines including IL-1β, IL-6 and TNF-α [5, 7]. Cascade release of inflammation aggravated fibrosis, microvascular and myocardial dysfunction. Immunological regulation of inflammation by exosomes further ameliorated myocardial injury and fibrosis [17]. Animal experiments pointed out that exosomes derived from atto vastatin (ATV)-pretreated MSCs facilitated angiogenesis and suppressed abnormal elevated TNF-α and IL-6 in peri-infarct region [49]. Moreover, miR-126 in exosomes obtained from adipose tissue-derived stem cells (ADSCs) was demonstrated to lower pro-inflammatory IL-1β, IL-6, and TNF-α as well as reduce MI [50]. In our study, we found that exosomes different from previous findings, derived from human PMSC, could suppressed pro-inflammatory IL-1β, IL-6, TNF-ɑ and MCP-1, suggesting that the effectiveness of PMSC-Exos intervention on MI may depend on suppressing the myocardial and circulating inflammation. Due to the critical role of macrophage in the inflammation, we consider that this benefit of PMSC-Exos on MI may probably correlate with the regulation of macrophage polarization via inhibiting M1 and promoting M2 subsets. Previous studies demonstrated pro-inflammatory M1 macrophages aggravated the progression of MI, whereas M2 with an anti-inflammation role could delay exacerbation of adverse LV remodeling [51]. Recent studies have found that exosomes as an immunomodulatory role in suppression of macrophage-induced inflammation [52]. Thus, the mechanism of macrophage polarization in the treatment with PMSC-Exos in MI is ongoing to be further investigated.

Accelerating evidences have demonstrated that gut microbiota is closely associated with the occurrence and development of MI [9, 53]. A clinical study found that AMI patients with significant increases in *Megasphaera*, *Butyricimonas*, *Acidaminococcus*, and *Desulfovibrio*, while decreases in *Tyzzerella*, *Dialister*, *Eubacterium ventriosum*, *Pseudobutyrivibrio*, and *Lachnospiraceae ND3007* in comparison with the healthy controls [53]. Consistently, we found that significant differences were presented between model group and sham group with regard to the fecal microbiome. Regarding the phylum level of gut microbiota, the abundance of *Bacteroidetes* and *Firmicutes* are the two most abundant phyla inhabiting the intestinal tract, of which the balance can be either beneficial or problematic to human and animal health, in consistency with previous studies [9]. *Firmicutes* play a vital role in absorbing calories from the diet and storing fat [33]. In addition, *Bacteroidetes* were reported to be implicated in immune regulation of inflammation [53]. Our results found that the abundance of *Firmicutes* was increased, while in *Bacteroidetes* was decreased in MI, indicating that the balance of gastrointestinal micro-ecological has been disrupted [53]. In further differences at the genus level, the model group was characterized by higher levels of *unclassified_Lachnospiraceae*, *Enterorhabdus* and *Desulfovibrio*, as well as lower levels of *Akkermansia*, *Alloprevotella* and *Ruminococcaceae*, which were supported by previous reports [53]. Interestingly, PMSC-Exos modulated gut microbiota in MI by up-regulating the abundances of probiotics *Akkermansia*, *Bacteroides*, *Bifidobacterium*, *Thauera* and *Ruminococcaceae*. *Bifidobacterium*, a probiotic known with an anti-inflammatory property, can directly attenuate inflammatory markers, cardiac injury and post-MI remodeling [54], although the mechanisms of cardioprotection are largely unknown. *Akkermansia* as the mucin-degrading bacterium, has been shown to ameliorate metabolic endotoxemia-induced inflammation through restoration of the gut barrier [55]. *Bacteroide*s were demonstrated to assist intestinal equilibrium by maintaining the diversity of gut microbiota and relieve inflammation by either modulating inflammatory cytokine production, which provided scientific supports for discovering next-generation probiotics [56]. Similarly, mesenchymal stem cell (MSC)-derived exosomal miR-181a alleviate experimental colitis model via affecting the gut microbiota composition, promoting intestinal barrier function and exerting anti-inflammatory function [57]. Taken together, we speculate that PMSC-Exos treatment may ameliorate the inflammation via elevating beneficial gut microbes, which contributes to providing potential targets for the MI treatment. However, the exact mechanism of Exos amelioration on MI by regulating gut microbiota remains unclear and needs to be further investigated.

Clinical studies showed that LPS translocation was closely associated with dysbacteriosis and inflammation in patients with MI [58]. In the experimental MI, compromised left ventricle (LV) function and intestinal hypoperfusion drove gut permeability elevation through suppression of tight junction protein and injury of intestinal mucosa [58]. Translocated LPS derived from intestinal pathogenic bacteria binds to TLR-4 to induce inflammatory cascade reaction [59]. In our study, the notable decrease of plasma LPS after PMSC-Exos treatment consistently supported the anti-inflammation role of PMSC-Exos in MI. We speculated that the decrease of LPS may be due to the rectification of gut dysbiosis in MI after PMSC-Exos treatment.

Additionally, gut microbiota carbohydrate metabolites SCFAs may contribute to maintaining host immune composition and repair capacity after MI [60]. This suggests that manipulation of these elements may provide opportunities to modulate pathological outcome after MI. Emerging studies have demonstrated that SCFAs can regulate the growth of gut microbial community and provide energy for gut microbiota and gut barrier cells of host [15]. Moreover, SCFAs also can suppress inflammation through GPRs-mediated pathway activation and HDAC suppression. Recently, animal studies have demonstrated that SCFAs (propionate acid, butyrate acid) are vital for cardiac repair capacity in MI mice with or without antibiotics [60]. Our results demonstrated that abnormal lower SCFAs (acetic acid, propionate acid, isobutyrate acid, butyrate acid, isovaleric and valeric acid) in MI model could be rectified after PMSC-Exos administration, indicating that SCFAs metabolism may participate in the effectiveness of PMSC-Exos. However, the mechanisms by which gut microbiota and related-metabolism can be released to the circulation upon the occurrence of cardiac infarction remains unclear. We surmise that modulation of SCFAs by PMSCs-Exos may probably via GPR pathway in inflammatory monocyte-macrophage or other immune cells such as regulatory T cells (Tregs), innate lymphoid cells (ILC), T helper cell 17 (Th17), neutrophils, and γδT cells, which still needs to be further determined.

Eventually, the close correlations among differential gut microbiota, inflammation and intestinal microbial SCFAs revealed that the gut microbiota-inflammation axis may play an important role in the pathogenesis and development of MI. Furthermore, protective PMSC-Exos on MI was probably due to the regulation of this critical axis.

Taken together, our results provide the first evidence that PMSC-Exos ameliorate MI via anti-inflammation and modulating gut microbiota, which contribute to integral understanding of pathogenesis of MI and potential application of PMSC-Exos for the control of the disease. However, the mechanism by which exosomes improve inflammation, gut microbiota and related-metabolites in MI remains largely unclear.

Exosomes have been thought to be critical carriers of biological information for facilitating intercellular communication. In this study, to further identify the definite composition molecules in PMSC-Exos which were responsible for the total or partial benefits in MI, whole transcription sequencing and analysis were performed. Based on the preliminary results and previous findings [19, 23], we speculated the most differential miRNAs were crucial in PMSC-Exos. MiRNAs play an important role in biological function including morphogenesis, epigenetic regulation, immune regulation, genesis and development of tumor [61]. An animal study found that human liver stem cell-derived microvesicles may inhibit hepatoma growth and stimulate apoptosis in severe combined immunodeficiency (SCID) mice by delivering antitumor miRNAs [62]. Exosomal transfer of miRNAs from the bone marrow may promote breast cancer cell dormancy in a metastatic niche [63]. Moreover, a finding indicated that differential expression of miRNA-127-3p, miRNA-409-3p, miRNA-410-3p, miRNA-541-5p and miRNA-540-5p may result in the destruction of inflammation pathways in sicca syndrome mice [64]. Additionally, Exosomes miR-18b derived from cardiogenic cells improved ischemia–reperfusion injury in MI rats via inducing macrophage activation [65]. Similarly, animal studies have found that exosomes (miR-19a-3p) ameliorated vascularization, decreased myocardial fibrosis, and increased LV ejection fraction as shown by transthoracic echocardiography in MI [66]. However, the specific mechanisms of miRNAs in beneficial effects on PMSC-Exos remain unknown and need to be addressed in further research.

## Conclusion

PMSC-Exos shows great advantages in anti-myocardial fibrosis, anti-inflammation, as well as modulation of gut microbiota, contributing to novel approaches and target for the control of MI.

## Data Availability

The datasets used and analysed during the current study are available from the corresponding author on reasonable request.

## Abbreviations

MI: Myocardial infarction
PMSCs: Placental mesenchymal stem cells
PMSC-Exos: Exosomes derived from human PMSCs
TEM: Transmission electron microscopy
NTA: Nanoparticle tracking analysis
LPS: Lipopolysaccharide
SCFAs: Short chain fatty acids,
TAL: Tachypleus amebocyte lysate
GC–MS: Gas chromatography mass spectrography
LV: Left ventricular
IL: Interleukin
TNF-α: Tumor necrosis factor-α
MCP-1: Monocyte chemoattractant protein-1
CVD: Cardiovascular disease
BMSCs: Bone mesenchymal stem cells
DMEM: Dulbecco’s modified eagle medium
FBS: Fetal bovine serum
PBS: Phosphate buffered saline
LAD: Left anterior descending
LEDd: Left ventricular end diastolic dimension
RVDd: Right ventricular end diastolic dimension
LVEF: Left ventricular ejection fraction
LVFS: Left ventricular fractional shortening
LVEDV: Left ventricular end-diastolic volume
RVEDV: Right ventricular end-diastolic volume
LVPWd: Left ventricular posterior wall diastolic thickness
TC: Total cholesterol
HDL-C: High density lipoprotein cholesterol
AST: Aspartate transaminase
BNP: Brain natriuretic factor or peptide,
MYO: Myoglobin
Tn-I: Troponin-I
ELISA: Enzyme linked immunosorbent assay
SIM: Single ion monitoring
PCA: Principal component analysis
PCoA: Principal coordinates analysis
NMDS: Nonmetric multidimensional scaling
OTU: Operational taxonomic unit
DAMPs: Damage-associated molecular patterns
GPRs: G protein-coupled receptors,
HDAC: Histone deacetylase
ECG: Electrocardiogram
SCID: Severe combined immunodeficiency
TMB: Tetramethylbenzidine
